# Accuracy of the strong‐ion gap: Dependency on albumin electrical charge and fluid electroneutrality

**DOI:** 10.14814/phy2.71006

**Published:** 2026-07-22

**Authors:** Matthew B. Wolf

**Affiliations:** ^1^ Department of Physiology, Pharmacology and Neuroscience University of South Carolina School of Medicine Columbia South Carolina USA

**Keywords:** acid–base balance, albumin electrical charge, electroneutrality constraint, mathematical model, strong‐ion gap

## Abstract

Figge's pH‐dependent approximation for albumin's electrical charge for native albumin has been widely used in acid–base analysis since 1992. Fogh‐Andersen (FA) subsequently demonstrated that albumin binds ions, primarily Cl^−^, increasing its net negative charge. A pH‐dependent equation for ion‐bound albumin was derived and compared with the Figge formulation for calculating the strong ion gap (SIG) using the electroneutrality relationship. Model performance was evaluated using datasets from studies of normal blood, where SIG is expected to be near zero. The primary dataset from Krbec et al. included over 300 measurement sets on blood from healthy subjects exposed to wide variations in PCO_2_, with comprehensive electrolyte and acid–base measurements. Across multiple data sets, FA model SIG values were consistently closer to zero than those using the Figge model. Analyses of three additional normal datasets supported this finding, whereas results from one study lacking Mg^2+^ measurements were less conclusive. In one of three clinical data sets, both formulations predicted SIG estimates that were clinically significant. The FA formulation appears preferable in the normal data sets studied, but further validation is needed before broad clinical replacement of the Figge formulation is warranted.

## 1 INTRODUCTION

For several decades, efforts have focused on developing methods to estimate the magnitude of unidentified or unmeasured anions in plasma. As reviewed by Morgan (2009), early applications of Stewart's strong‐ion difference (SID) framework (Stewart, 1981), initially by Jones (1990), defined this difference as the net electrical charge primarily attributable to plasma proteins. Kellum et al. (1995) and Kellum et al. (2009) later extended this concept by introducing the strong‐ion gap (SIG), representing the net charge of all unmeasured ions excluding inorganic phosphate (Pi), which can be independently quantified. Compared with the traditional anion gap (AG), defined by Emmett and Narins (1977) as a simplified approximation of the plasma electroneutrality equation, SIG was shown (Kellum et al., 1995) to be a more accurate predictor of unmeasured ions. The term SIG was subsequently replaced by net unidentified ions (NUI) in computational implementations designed to calculate acid–base variables (Elbers et al., 2015).

Morgan (2009) emphasized the need for improved methods to detect unmeasured ions and proposed SIG as a more reliable alternative to approaches such as the albumin‐corrected anion gap (AGc), noting the absence at that time of direct comparative evaluations. This gap concept was later addressed by Morgan et al. (2016), who performed a head‐to‐head comparison using blood samples from patients undergoing cardiopulmonary bypass, in which acetate and gluconate concentrations present in the bypass fluids were directly measured. Multiple diagnostic tools, including SIG, were assessed alongside predictions from multicompartment computational models developed by Anstey (2010) and by Wolf and DeLand (2011). While these models demonstrated the highest predictive accuracy, SIG ranked closely behind and was identified as the most practical clinical tool due to its relative simplicity.

The accuracy of SIG estimation depends on a number of key assumptions: plasma electroneutrality, accurate quantification of albumin charge and inclusion with precise measurement of all required electrolytes and other acid–base variables. Ring and Kellum (2016) demonstrated that electroneutrality‐based approaches can reliably predict blood pH across a range of pathological states, assuming the validity of the Figge–Rossing–Fencl (Figge) model for albumin charge (Figge et al., 1991; Figge et al., 1992) as reformulated by Watson (1999). However, the Ring and Kellum (2016) formulation required an additional empirical term, suggesting that important charge interactions may not have been fully taken into account.

Although the Figge model accurately describes the charge of native albumin and reproduces measured pH‐dependent charge data (Figge 2009), it does not incorporate ion‐binding effects. Fogh‐Andersen et al. (1993) demonstrated that albumin binds ions—particularly Cl^−^ and Ca^2+^—in a pH‐dependent manner, resulting in a more negative net charge than predicted for native albumin alone. Neglecting these interactions may introduce systematic error into SIG calculations and related acid–base analyses.

An important consideration in evaluating the accuracy of SIG is that, even under ideal conditions (i.e., in the absence of electrolyte measurement error), its normal value is less than 2 mEq/L and is generally considered to approach zero (Kellum et al., 2009). Under normal acid–base conditions, the negative charge attributed to albumin is estimated to be approximately 4 mEq/L greater when calculated using the albumin–ion binding data of Fogh‐Andersen et al. (1993) compared with the charge of native albumin alone. Consequently, distinguishing between the magnitudes of these charge approximations on SIG estimations with certainty is inherently challenging.

Anstey (2009) used the electroneutrality equation with a complete set of required measurements and the pH‐dependent, Figge albumin charge relationship (Figge et al., 1992) to determine SIG distribution of a general volunteer hospital population by using a Monte Carlo simulation. He found a mean value of 3.9 mEq/L, whereas the expected value is near zero. He dismissed the significance of measurement errors but suggested that the albumin ion‐binding effects found by Fogh‐Andersen et al. (1993) could explain his findings.

The present study applies plasma electroneutrality models to both experimental and clinical data bases. Two approximate, pH dependent formulations for albumin charge are evaluated: the standard one of Figge et al. (1992) and a new version incorporating ion‐binding effects based on Fogh‐Andersen et al. (1993). Experimental data from Krbec et al. (2022), comprising extensive individual measurements of acid–base variables across a wide range of PCO_2_ values in normal and acidotic blood, form the primary basis for model assessment. Additional validation is provided using mean data from various published studies on human blood and mean patient data from clinical studies.

The first objective of this study is to use the Fogh‐Andersen et al. (1993) data to determine a pH‐dependent albumin electrical charge formulation to see which albumin charge formulation more accurately estimates SIG using measurement data from a number of independent studies, where expected values approach zero. The second objective is to compare SIG predictions from both formulations in clinical settings, including a case where concentrations of typically unmeasured ions were directly measured and ones where measurements were made to use SIG estimates to categorize various acid–base abnormalities in clinical studies.

Ultimately, this work aims to identify the most appropriate albumin charge formulation for use in both experimental studies of normal blood and clinical acid–base analyses.

## 2 METHODS AND MATERIALS

### 2.1 Determination of albumin–ion binding relationship

**FIGURE 1:**
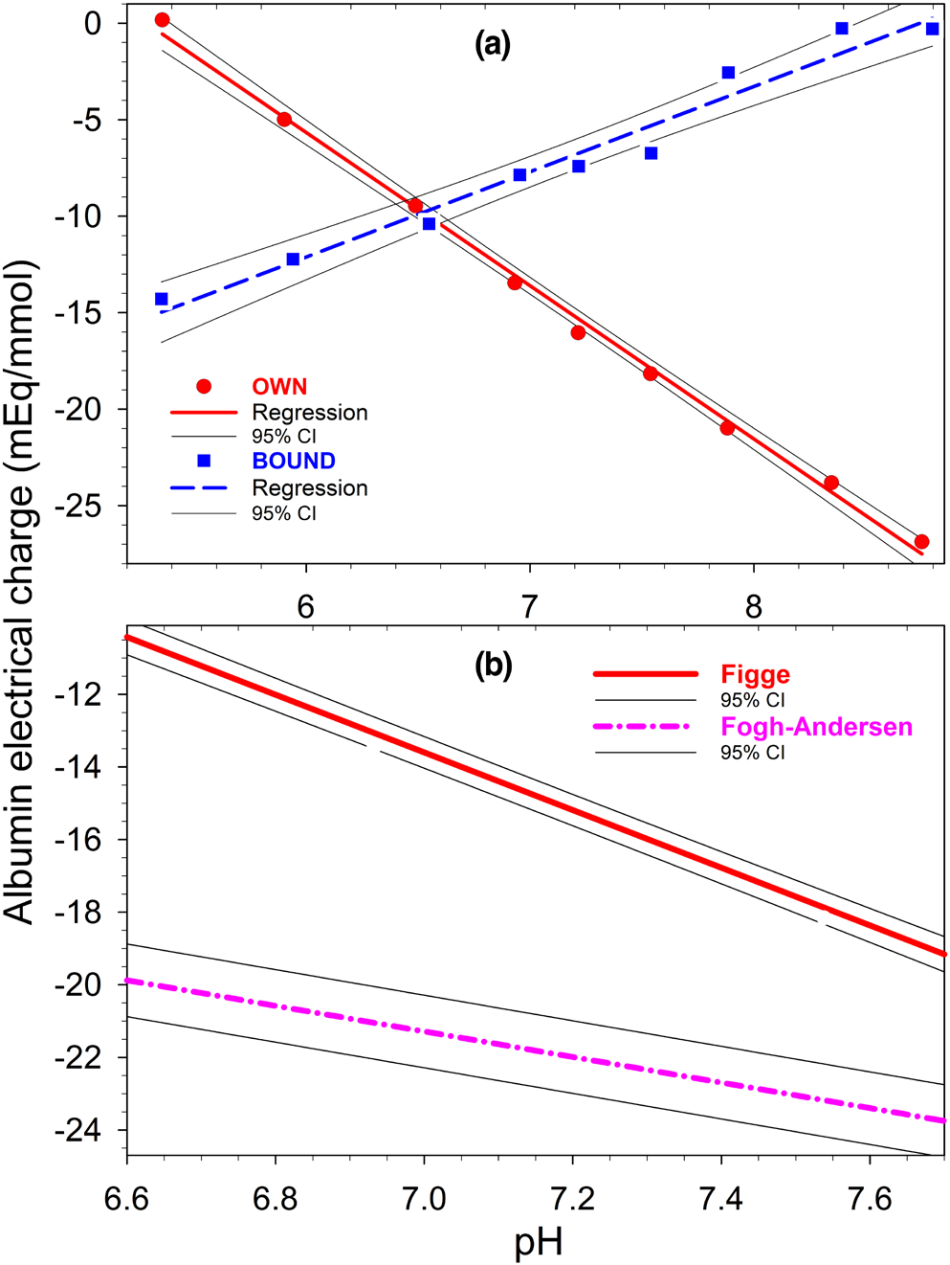
(a) Albumin's pH‐dependent OWN charge data (red solid‐filled circles) and BOUND charges (blue solid‐filled circles) were captured over a wide pH range using a Graph Digitizer program. Regression lines, blue and red, respectively, with 95% confidence intervals (CI), were determined for both sets with statistics given in Table 1. (b) Over a narrower, physiological pH range, the pink dashed line with 95% CIs shows the sum of the intercepts and slopes of the two regression lines in 1a. The equation for this line, shown as Equation 1 in more commonly used units, is designated as the pH‐dependent, Fogh‐Andersen albumin electrical charge relationship. Equation 2 describes a repeat of the OWN regression line (red) over this narrower pH range. It is equivalent to the Figge charge relationship.

Data from Fogh‐Andersen et al. (1993) were used to characterize the pH‐dependent electrical charge (mEq/mmol) of native albumin (OWN) and albumin‐bound ions (BOUND), primarily Cl^−^ and Ca^2+^, as illustrated in Figure 1a. A broad pH range of approximately 5–9 was analyzed because the relationships appeared linear throughout this interval. Albumin intrinsic charge (red solid circles) and bound‐ion charge (blue solid circles) were digitized using GetData Graph Digitizer (GetData Software, Kogarah, NSW, Australia). The blue and red lines represent linear regressions of the BOUND and OWN data, respectively, with adjacent narrow 95% confidence intervals (CI; black lines). Table 1 shows intercept and slope statistics of the two regression lines, each with relatively small SE values and almost unity coefficients of determination (*R* ^2^).

**TABLE 1:** Regression fits to Fogh‐Andersen albumin electrical charge data (Figure 1).

Albumin electrical charge	Intercept	Slope	*R* ^2^
	mEq/mmol	mEq/L/mmol/mmHg	
Own	42.01 ± 1.256 (SE)	−7.944 ± 0.174	0.997
Bound	−38.64 ± 2.28	4.421 ± 0.314	0.966
Sum	3.37 ± 3.46	3.523 ± 0.359	

*Note*: Assuming an albumin molecular weight of 66.5 g/mmol, the summed intercept and slope values are 0.0506 mEq/g and 0.053 mEq/g/mmHg respectively (See Equation 1).

Abbreviation: *R* ^2^, coefficient of determination.

Figure 1b shows the regression (pink dashed line) for the combined (summed) charge contribution (OWN + BOUND), derived from the two regressions in Figure 1a, together with its somewhat broader 95% CI, which does not overlap the OWN regression line. Table 1 shows that the summed intercept of 3.37 mEq/L has a relatively large SE of 3.46 due to its back‐extrapolation to zero pH, but more importantly, for present purposes, the slope is 3.523 mEq/mmol/mmHg with a relatively small 0.359 SE value. Assuming a molecular weight of 66.5 g/mmol for albumin, the summed intercept and slope values are 0.0506 mEq/g and 0.053 mEq/g/mmHg, respectively as given in Equation 1. This result is designated as the new Fogh‐Andersen (FA) albumin charge formulation (model).

The OWN regression (blue) repeated in Figure 1b is the present pH‐dependent native albumin charge model. When its intercept and slope given in Table 1 are converted into conventional units, it is almost identical to Equation 2 found by Figge et al. (1992), hence that equation will be used as the present Figge albumin charge model.

(1) $$Z_{\mathrm{Alb}}^{\mathrm{FA}}\;\left(\mathrm{mEq}/g\;\mathrm{Alb}\right)=0.0506-0.053\times \mathrm{pH}$$

(2) $$Z_{\mathrm{Alb}}^{\text{Figge}}\;\left(\mathrm{mEq}/g\;\mathrm{Alb}\right)=0.631-0.123\times \mathrm{pH}$$

At normal pH (7.4) and albumin concentration of 44 g/L, Equation 1 predicts approximately 4 mEq/L more negative charge than Equation 2, with the difference increasing further within the clinically important metabolic acidosis range.

### 2.2 Electroneutrality models

The apparent strong‐ion difference (SIDa) is defined as:

(3) $$\;\text{SIDa}=\left[Na^{+}\right]+\left[K^{+}\right]+2\times \left[\mathrm{ICa}\right]+2\left[Mg^{2+}\right]-\left[Cl^{-}\right]-\left[\mathrm{La}c^{-}\right]$$

where ICa is ionized calcium, Lac is lactate and all concentrations are expressed in mmol/L (mM); SIDa is in mEq/L. Ionized calcium (ICa) is used where available. If only total calcium is measured, ICa is estimated as:

(4) $$\left[\mathrm{ICa}\right]=\frac{\left[\mathrm{Ca}\right]\times 39.8}{\left[H^{+}\right]+39.8}$$

where $\left[H^{+}\right]$ is expressed in nmol/L.

The effective strong‐ion difference (SIDe) is given by:

(5) $$\text{SIDe}=\left[\mathrm{HC}O_{3}^{-}\right]+\left[Pi^{-}\right]+\left[\mathrm{Al}b^{-}\right]$$

where all terms are expressed in mEq/L. The charge contribution of inorganic phosphate (Pi) is approximated by:

(6) $$\left[Pi^{-}\right]=\left[\mathrm{Pi}\right]\times \left(0.309\times \mathrm{pH}-0.469\right)$$

with $\left[\mathrm{Pi}\right]$ in mmol/L.

Albumin charge $\left[\mathrm{Al}b^{-}\right]$ is calculated by multiplying albumin concentration (g/L) by either Equation 1 or Equation 2.

The strong‐ion gap (SIG) is defined as follows:

(7) SIG=SIDa−SIDe

Both SIDa and SIDe are relatively large numbers; hence small errors in their determination can lead to large errors in SIG.

For a complete and error‐free dataset, the net unidentified ions (NUI) parameter is equivalent to SIG.

### 2.3 Normal blood experimental data

In the following studies, SIG values were determined using both Equations 1 and 2 and (3), (4), (5), (6), (7) with the basic measured data. All experimental data and SIG calculations are provided in Excel format, Data S1–S6 https://doi.org/10.6084/m9.figshare.32325519.

Krbec et al. (2022) reported more than 300 measurements from 11 individual human blood samples, including pH, independently controlled PCO_2_, and corresponding plasma concentrations of Na^+^, K^+^, ICa, Cl^−^, HCO_3_^−^, and lactate. Albumin, Mg^2+^ and Pi concentrations were measured once per sample prior to extensive PCO_2_ manipulation. Measurements were obtained under three conditions: control (CTR), Cl 7.5, and Cl 15.

Control blood was diluted 1:25 with a solution designed to maintain near‐normal Na^+^ and Cl^−^ levels. Additional HCl was added to increase plasma [Cl^−^] by approximately 7.5 or 15 mEq/L, producing the Cl 7.5 and Cl 15 conditions.

Rossing et al. (1986) performed similar measurements on five normal blood samples. Lactate was not measured in these experiments. Fencl et al. (2000) conducted comparable studies on nine normal blood samples, but lactate concentrations were not reported. To estimate the statistical distribution of SIG in a general population, Anstey (2009) used a complete set of biochemical data obtained from the AUSLAB hospital information system.

Morgan et al. (2007) measured biochemical variables in 37 normal blood samples across four experimental groups to assess consistency in SIG calculations. Two groups consisted of undiluted blood at extreme PCO_2_ levels, while the other two included sodium lactate supplementation under similar conditions. Magnesium concentrations were not measured.

### 2.4 Clinical cases

Morgan et al. (2016) reported electrolyte and acid–base measurements at four time points in patients undergoing cardiopulmonary bypass. The priming solution contained 50–52 mEq/L of acetate and gluconate, which are not routinely measured. Magnesium concentrations were not reported even though it was present in the bypass fluids. At each time point, SIG was estimated using both formulations to compare with measured acetate plus gluconate values.

Two studies by Rocktaeschel, Morimatsu, Uchino, Goldsmith, et al. (2003) and Rocktaeschel, Morimatsu, Uchino, & Bellomo (2003) report acid–base measurements in critically ill patients required for estimation of SIG from the electroneutrality equation as well as other acid–base scanning tools. The first study (Rocktaeschel, Morimatsu, Uchino, Goldsmith, et al., 2003) is with acute renal failure (ARF) patients and compares their SIG values with matched non‐ARF patient controls as well as non‐ARF ICU controls. The second study (Rocktaeschel, Morimatsu, Uchino, & Bellomo, 2003) determines unmeasured ions (SIG) in both surviving and non‐surviving patients to see if SIG values can predict mortality.

The final clinical study is by Kroustalakis et al. (2025) who made complete sets of measurements to determine SIG and other acid‐base scanning tool values in a group of RF patients before and after Hemodialysis (HD) and in another group of Peritoneal dialysis (PD) patients. Their purpose was to compare these prediction using both the Stewart (1981) and the traditional Henderson‐Hasselbalch (HH) analytical models. Although this study was quite interesting from a general acid‐base diagnostic aspect, for present purposes, only the data relative to SIG determinations were analyzed.

## 3 RESULTS

The data shown in each of the Tables were derived from original data and calculations given on Excel spreadsheets in Data S1–S6.

**FIGURE 2:**
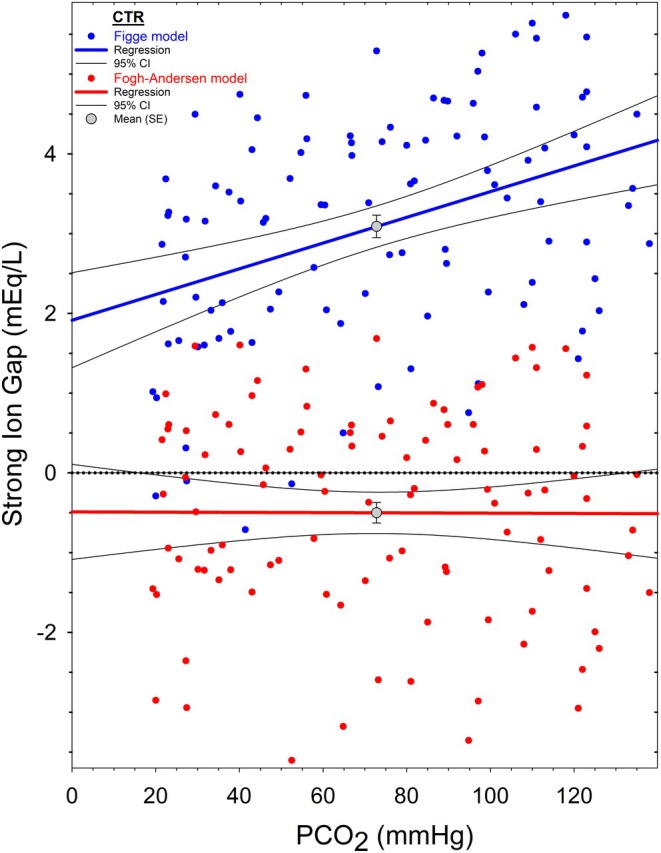
Individual Fogh‐Andersen (FA) model (Equation 1) SIG estimations using Krbec et al. control (CTR) measurement data are shown as solid blue circles and individual SIG estimations using Figge albumin charge model (Equation 2) are shown as solid red circles. Blue and red lines are linear regressions with 95% CIs (black lines) to these data. Also shown are mean values and SE bars (gray circles) for each model CTR estimation. See Tables 2 and 3 for statistical analyses.

**FIGURE 3:**
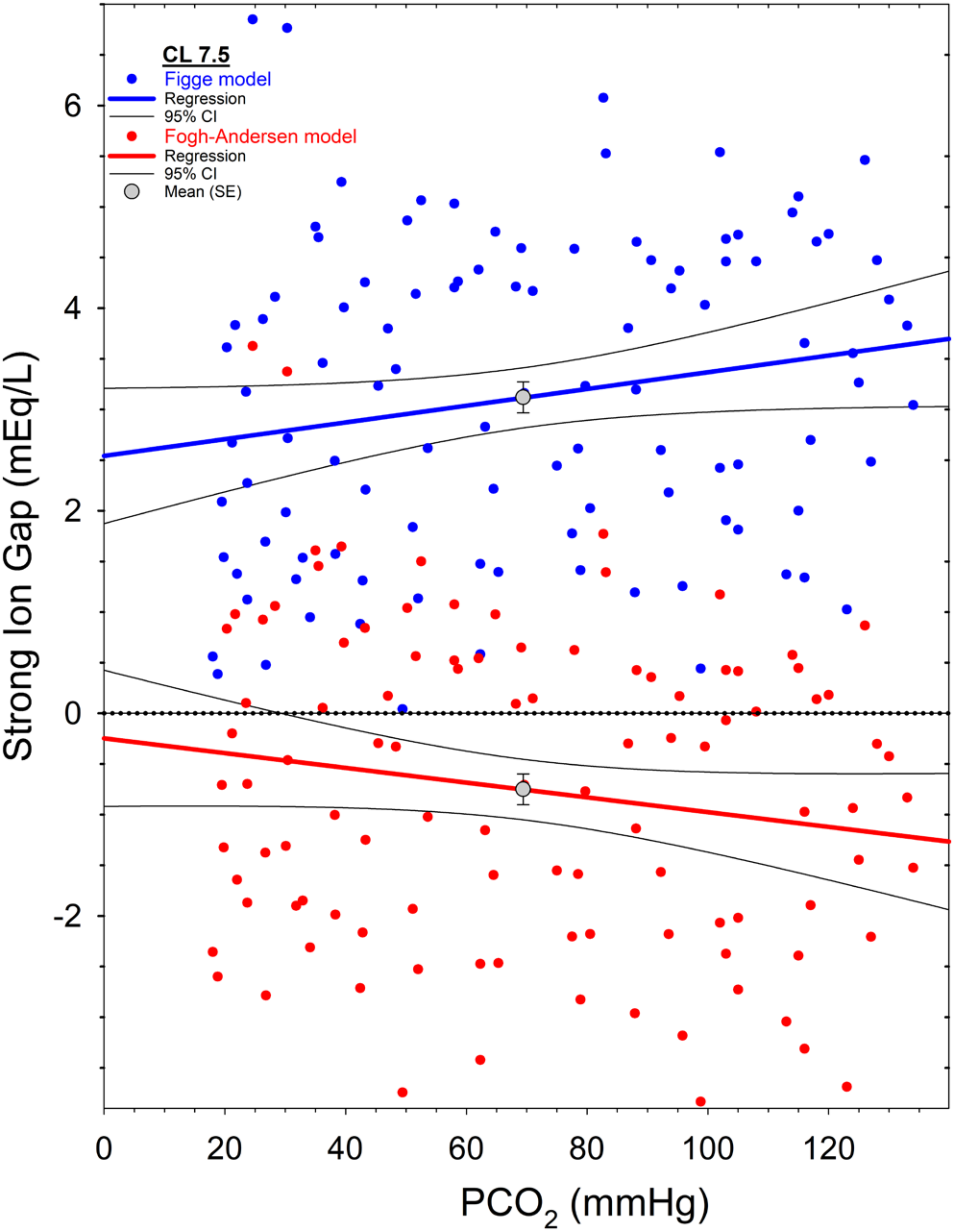
Same as Figure 2, except that estimations were for CL 7.5 experiments.

**FIGURE 4:**
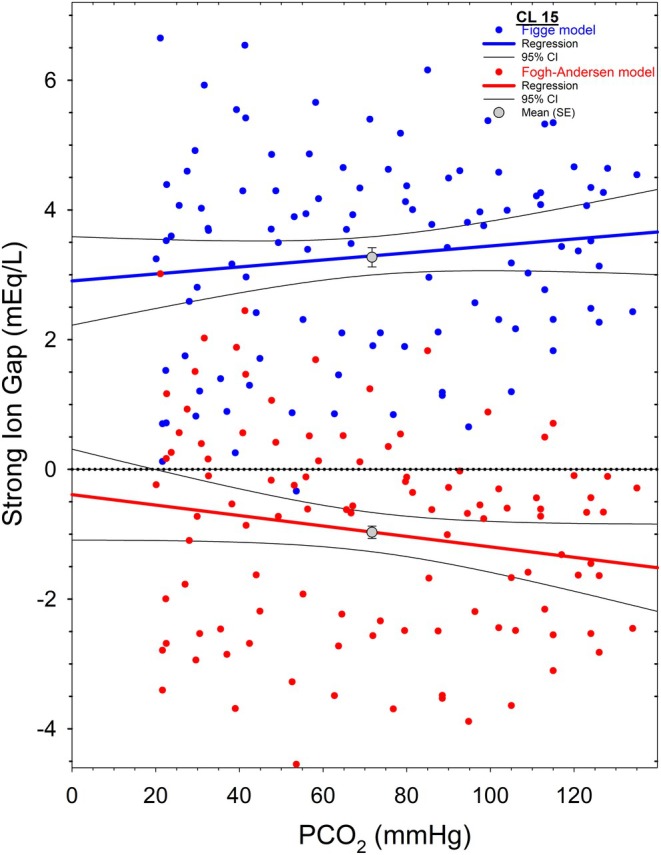
Same as Figure 2, except that estimations were for CL 15 experiments.

Equations (3), (4), (5), (6), (7), combined with Equation 1 or Equation 2, define the Fogh‐Andersen (FA) and Figge models, respectively, from which SIG values were calculated. Figures 2, 3, 4 present individual SIG estimates plotted against PCO_2_ values for Figge (red circles) and FA (blue circles) models across three experimental conditions reported by Krbec et al. (2022) (CTR, CL 7.5, and CL 15), along with matching linear regression lines, 95% CI bounds (black) and group means ± SE (gray circles).

Across all conditions, FA model estimates were consistently closer to the theoretical SIG value of zero with 95% CI bands non‐overlapping Figge model estimates. In the CTR dataset (*N* = 104), mean SIG was 3.19 ± 0.12 mEq/L (SE) for the Figge model compared with −0.37 ± 0.11 mEq/L for the FA model (Table 2). The Figge model exhibited a slope of 16 ± 3.7 × 10^−3^ mEq/L/mmHg (R^2^ = 0.16), whereas the FA model showed a near‐zero slope (0.16 ± 3.7 × 10^−3^ mEq/L/mmHg) with negligible *R* ^2^, indicating minimal dependence of SIG on PCO_2_. Similar findings were observed in the CL 7.5 and CL 15 groups, with mean SIG values of 3.23 and 3.4 mEq/L (Figge) compared to −0.61 mEq/L and −0.81 (FA).

**TABLE 2:** Comparison of Figge and Fogh‐Andersen albumin electric‐charge estimation models on strong‐ion gap (SIG) predictions. Group data from Krbec et al.

Experiment	*N*	SIG Figge model regression			SIG Fogh‐Andersen model regression		
		Mean values ± SE (mEq/L)	Slope ± SE × 10^−3^ (mEq/L/mmHg)	*R* ^2^	Mean values ± SE (mEq/L)	Slope ± SE (×10^−3^) (mEq/L/mmHg)	*R* ^2^
CTR	104	3.19 ± 0.12	16 ± 3.7	0.16	−0.37 ± 0.11	0.16 ± 3.7	0
CL 7.5	104	3.23 ± 0.13	8.3 ± 4.3	0.034	−0.61 ± 0.13	−7.3 ± 4.3	0.027
CL 15	106	3.4 ± 0.13	5.4 ± 4.3	0.015	−0.81 ± 0.13	−5.4 ± 4.3	0.015
Group mean		3.27			−0.6		

*Note*: See Data S1.

Abbreviations: *N*, number of observations; *R* ^2^, coefficient of determination; SIG, strong‐ion gap.

Table 3 shows individual subject level analyses. For Subject A ([Alb] = 49 g/L), the mean of 11 CTR measurements was 4.57 ± 0.88 mEq/L (SD) using the Figge model and 1.22 ± 0.36 mEq/L using the FA model. Comparable differences between models were observed across all subjects and experimental conditions, with FA‐derived SIG values consistently closer to zero. Group means across subjects were consistent with these findings; in the CTR condition, mean SIG was 3.19 ± 1.23 mEq/L (SD) for the Figge model and − 0.37 ± 1.08 mEq/L for the FA model, with similar differences in the CL 7.5 and CL 15 groups. See Data S1 for original data and Excel calculations.

**TABLE 3:** Comparison of Figge and Fogh‐Andersen albumin electric‐charge estimation models on strong‐ion gap (SIG) predictions. Individual subject data from Krbec et al.

Subject	SIG Figge model						SIG Fogh‐Andersen model		
([Alb]) g/L	CTR		CL 7.5		CL 15		CTR	CL 7.5	CL 15
	Mean values ± SD (mEq/L)						Mean values ± SD (mEq/L)		
	*N*		*N*		*N*				
A^a^ (49)	11	4.57 ± 0.88	12	4.26 ± 0.51	11	4.21 ± 0.49	1.22 ± 0.36	0.61 ± 0.8	0.18 ± 0.69
B^a^ (49)	11	2.61 ± 0.61	11	2.42 ± 0.84	12	3.85 ± 0.54	−0.82 ± 0.44	−1.38 ± 0.53	−0.26 ± 0.45
C^a^ (51)	11	3.85 ± 0.86	11	4.65 ± 1.4	11	4.51 ± 1.3	0.00 ± 0.61	0.6 ± 1.49	0.04 ± 1.68
D^a^ (51)	10	3.58 ± 1.21	11	4.07 ± 0.59	10	4.95 ± 0.59	0.1 ± 0.72	0.19 ± 0.74	0.82 ± 0.61
E^a^ (52)	10	3.21 ± 0.49	9	3.46 ± 0.62	10	3.88 ± 0.65	−0.59 ± 0.48	−0.48 ± 0.44	−0.49 ± 0.67
F (50)	10	4.47 ± 0.68	11	3.86 ± 1.51	11	3.98 ± 1.24	0.84 ± 0.78	0.13 ± 0.95	−0.3 ± 0.94
G (51)	10	1.4 ± 0.73	9	1.05 ± 0.52	10	1.47 ± 0.87	−2.31 ± 0.55	−2.81 ± 0.71	−2.81 ± 0.68
H (51)	11	2.75 ± 1.03	10	2.52 ± 0.76	11	2.3 ± 0.75	−0.86 ± 0.46	−1.53 ± 0.49	−2.12 ± 0.5
J (49)	10	2.63 ± 0.74	10	2.2 ± 0.92	10	2.35 ± 1.26	−0.86 ± 0.53	−1.66 ± 0.65	−1.78 ± 1.11
K (43)	10	2.54 ± 0.93	10	2.72 ± 0.51	11	2.58 ± 0.69	−0.63 ± 0.58	−0.73 ± 0.58	−1.41 ± 0.55
Mean ± SD	104	3.19 ± 1.23	104	3.23 ± 1.34	107	3.4 ± 1.33	−0.37 ± 1.08	−0.61 ± 1.31	−0.81 ± 1.35

*Note*: See Data S1.

^a^ PCO_2_ change sequence from high to low; [Alb], albumin concentration; *N*, number of observations (Same for both models): SIG, strong‐ion gap.

Table 4 summarizes mean SIG estimates from three additional datasets of normal subjects, all with normal [Alb]. For the Rossing et al. (1986) five subjects, the mean FA SIG prediction was −0.99 mEq/L compared to the 1.83 mEq/L Figge prediction. In the Fencl et al. (2000) data set of nine subjects, the mean FA SIG estimate of 0.84 mEq/L was much closer to zero than the 4.39 mEq/L Figge estimate.

**TABLE 4:** Comparison of two albumin‐charge estimation models on strong‐ion gap predictions in three individual studies.

Author	*N*	[Alb]	SIG Figge model	SIG Fogh‐Andersen model
		g/L	Mean values (mEq/L)	
Rossing et al.	5	47	1.83	−0.99
Fencl et al.	9	44	4.39	0.84
Anstey	^a^	42	4.12	0.58
Group mean			3.45	0.14

*Note*: See Data S2.

Abbreviations: *N*, number of observations: SIG, strong ion gap.

^a^ Not given.

In the Monte Carlo analysis of Anstey (2009), based on numerous hospital biochemical data, mean FA SIG estimates were 0.58 mEq/L compared to 4.12 mEq/L Figge estimates. Across all three datasets, mean FA model predictions were consistently much closer to zero than those of the Figge model. See Data S2.

A potential exception is the study by Morgan et al. (2007), which examined normal and sodium lactate–diluted blood under very low and high PCO_2_ conditions. Their reported SIG values (Table 5, marked with a) calculated with the Figge model (Equation 2) had an overall mean near zero, and the relatively large standard deviations suggest that individual means in all four conditions were not significantly different from zero. However, magnesium concentrations ([Mg]) were not measured.

**TABLE 5:** Comparison of SIG predictions using Morgan et al. (2007) normal blood measurements.

						Mg corrected		
PCO_2_ state		[Alb] g/L	*N*	Assumed [Mg] mM	Morgan measurements mean ± SD mEq/L^a^	Morgan measurements mEq/L	Figge model mEq/L	Fogh‐Andersen model mEq/L
	mmHg							
					Undiluted			
Initial	264	44	11	0.8	*−*1.2 ± 1.3	0.4	0.51	−3.16
Final	19	44	10	0.8	0.4 ± 1.5	2	1.99	0.7
					Diluted with Na Lactate solution			
Initial	232	24	9	0.44	−0.1 ± 1.9	0.78	0.25	−1.87
Final	15	24	7	0.44	0.8 ± 0.8	1.68	1.66	0.92
					Group mean			
					0.5 ± 1.5	1.22	1.1	−0.85

*Note*: See Data S3.

Abbreviations: *N*, number of observations; SIG, strong‐ion gap.

^a^ Used Figge albumin formulation (Equation 2).

Assuming a normal [Mg] of 0.8 mM would increase estimated SIG by approximately 1.6 mEq/L in undiluted samples and 0.88 mEq/L in diluted samples, yet recalculated values still remained within the 0–2 mEq/L range. Corrected Figge model predictions were consistent with these findings, as they also employ Equation 2. In contrast, FA model estimates were generally more negative but, aside from the undiluted high PCO_2_ condition, remained reasonably close to zero as shown by the group mean values. See Data S3.

### 3.1 Clinical cases

Table 6 presents strong ion gap (SIG; denoted XA) estimates for cardiac bypass patients reported by Morgan et al. (2016) at four procedural time points. Their original XA values (acetate and gluconate) were calculated using the Figge model. Using the same dataset, SIG values were recalculated here with both the Figge and FA models, while accounting for the absence of measured [Mg]. Revised Figge estimates were derived to maintain consistency with the originally reported values. Estimated [Mg] ranged from −0.84 mEq/L at T1 (pre‐bypass) to 4.7 mEq/L at T2 (2 min after bypass initiation), with intermediate values calculated at the remaining time points. Corresponding FA model estimates were slightly lower but showed a similar temporal pattern. For both models, SIG values were closest to zero at T1 (pre‐bypass) and T4 (4‐h post‐bypass). See Data S4.

**TABLE 6:** Comparison of Figge and Fogh‐Andersen model SIG estimations for cardiac‐bypass patients. Mean data from Morgan et al. (2016).

Measurement time	[Mg]^a^ added	Morgan et al. [XA] measurements	SIG model estimations	
			Figge	Fogh‐Andersen
	mEq/L	Mean ± SD (mEq/L)	mEq/L	
T1	−0.84	1.41 ± 0.87	1.41	−0.57
T2	4.7	11.73 ± 3.28	11.73	10.45
T3	2.6	4.8 ± 1.49	4.8	3.54
T4	1.26	1.36 ± 1.73	1.36	−0.52

*Note*: T1. Immediately before commencing cardio‐pulmonary bypass; T2. 2 min after commencement of bypass, prior to placement of the aortic cross‐clamp; T3. On rewarming, just prior to separation from bypass. *N* = 15 patient measurements at each time point; SIG, strong‐ion gap; [XA], measured concentrations of acetate and gluconate. See Data S4.

^a^ [Mg] added to match Figge model SIG estimations to measured [XA] mean values.

The authors of the next two clinical studies calculated strong ion gap (SIG) using the Figge formula (Equation 2); therefore, comparisons here were limited to recalculations performed with the same equation using their published measurement data.

The upper portion of Table 7 presents SIG estimates reported by Rocktaeschel, Morimatsu, Uchino, & Bellomo (2003) for patients with acute renal failure (ARF) and corresponding control groups. Their SIG values were derived from Equation 7 using the reported mean SIDa and SIDe values shown in the table. Using the Figge model, the reported SIG for the 40 ARF patients, whose mean [Alb] was markedly reduced at 22.6 g/L, was 13.4 ± 5.5 mEq/L, calculated from the difference between the mean SIDa value of 42.4 ± 4.4 mEq/L and the mean SIDe value of 29.0 ± 5.1 mEq/L. Recalculation in the present analysis using the same mean data yielded a nearly identical value of 13.7 mEq/L. Similar comparisons were obtained in both control groups, but the differences were somewhat larger.

**TABLE 7:** Comparison of Strong‐ion gap (SIG) predictions by Rocktaeschel et al. and present Figge model using same basic data.

	*N*	[Alb] g/L	Rocktaeschel			Figge		
			SIDa	SIDe	SIG	SIDa	SIDe	SIG
			Means ± SD (mEq/L)					
Experiment								
Acute renal failure	40	22.6	42.4 ± 4.4	29 ± 5.1	13.4 ± 5.5	42.4	28.6	13.7
Matched controls	40	25.2	42.8 ± 4.4	33.4 ± 6.3	9.5 ± 4.4	42.8	32.8	10
ICU controls	60	23.9	45.2 ± 3.7	36.9 ± 5.5	8.3 ± 3.6	45.2	36.3	8.9
Survivors								
Median (mEq/L)	217	27	43.5	35.4	7.8	44.3	34.5	9.79
Mean (mEq/L)^a^	217	27	43.4	35.5	7.94 ± 4.03	44.2	34.6	9.65
Non‐survivors								
Median (mEq/L)	83	25	43.6	32.7	9.5	43.5	32.5	11.1
Mean (mEq/L)^a^	83	25	43.8	33.2	10 ± 4.23	43.9	32.5	11.3

*Note*: See Data S5.

Abbreviations: *N*, number of observations; *N*, number of patients; SIDa, apparent strong ion difference (Equation 3); SIDe, effective strong ion difference (Equation 5) SIG, strong ion gap (Equation 7).

^a^ Calculated from statistical methods in medical research, Luo, D. et al. 27(6) 1785–1805.

The lower portion of Table 7 summarizes a second study by Rocktaeschel, Morimatsu, Uchino, Goldsmith, et al., 2003; Rocktaeschel, Morimatsu, Uchino, & Bellomo, 2003, in which median SIG values calculated with Equation 2 were reported for critically ill survivors and non‐survivors. Median values were converted to approximate means using the online calculator referenced in the table. For the 217 survivors, the reported median SIG was 7.8 mEq/L, closely matching the recalculated mean value of 7.94 mEq/L. For the 83 non‐survivors, the reported median SIG of 9.5 mEq/L was similarly close to the recalculated value of approximately 10 mEq/L; however, present calculations with the Figge model yielded values 1–2 mEq/L greater than the calculations mainly because of slightly higher SIDa values and slightly lower SIDe ones than their calculations. See Data S5.

Table 8 presents the SIG estimates (Equation 7) reported by Kroustalakis et al. (2025), calculated from SIDa (Equation 3) and SIDe (Equation 5), for renal failure (RF) patients before hemodialysis (pre‐HD), after hemodialysis (post‐HD), and during peritoneal dialysis (PD). These values are compared with SIG predictions generated using the Figge model (Equation 2) and the FA model (Equation 1). For the pre‐HD and post‐HD measurements, the present Figge model predictions closely match the values reported by Kroustalakis et al. (2025). As expected, the FA model, which assigns a greater negative charge to albumin, yields SIG estimates approximately 2 mEq/L lower than those obtained with the Figge model. Kroustalakis et al. (2025) reported that pre‐HD SIG values were significantly higher than both the post‐HD and PD values.

**TABLE 8:** Comparison of strong‐ion gap (SIG) predictions by Kroustalakis et al. and present models using the same basic data.

Experiment	*N*	[Alb]	Kroustalakis			Models			
						Figge			FA
			SIDa	SIDe	SIG	SIDa	SIDe	SIG	SIG
		g/L	mEq/L						
Pre‐HD	53	39.5	41 ± 2.54	33.2 ± 2.72	7.82 ± 2.88	41	33.2	7.8	5.1
Post‐HD	53	41.3	42.4 ± 1.96	38.5 ± 1.64	3.94 ± 2.27	42.4	38.5	3.92	1.42
PD	41	33.1	44.3 ± 3.64	35.6 ± 3.44	8.7 ± 3.1	44.3	37.6	8.74	4.63

*Note*: See Data S6.

Abbreviations: HD, hemodialysis; *N*, number of patients; PD, peritoneal dialysis; SIDa, apparent strong ion difference (Equation 3); SIDe, effective strong ion difference (Equation 5); SIG, strong ion gap (Equation 7).

## 4 DISCUSSION

The present study proposes an alternative to the commonly used albumin electrical charge model of Figge et al. (1991) and Figge et al. (1992), which underpins most strong‐ion gap (SIG) calculations and related acid–base analyses, including clinical pH predictions. The alternative formulation is based on data from Fogh‐Andersen et al. (1993) and incorporates both the intrinsic charge of albumin and the contribution of bound ions, primarily Cl^−^ and Ca^2+^.

Both intrinsic and ion‐bound charge datasets were well described by linear relationships with pH (Figure 1, Table 1). Combining these relationships, but changing to conventional units, yielded a single pH‐dependent expression (Equation 1) that accounts for ion‐binding effects. This contrasts with the Figge formulation (Equation 2), which represents only the intrinsic charge of albumin.

Accurate estimation of SIG depends not only on the albumin charge approximation, but also on the completeness of the electroneutrality framework and the accuracy of measured variables. In this study, all routinely measured ions (Na^+^, K^+^, Ca^2+^, Mg^2+^, Cl^−^, HCO_3_^−^, Lac^−^, and Pi in mM concentrations) were included when reported. Because bicarbonate is often derived using the Henderson–Hasselbalch equation with variable constants, reported values from the original studies were used directly to minimize methodological variability.

Across the datasets examined, the Fogh‐Andersen (FA) formulation (Equation 1) consistently yielded SIG values closer to the expected value of zero than the Figge formulation (Equation 2). This was most clearly demonstrated in the large dataset of Krbec et al. (2022), where over a wide range of PCO_2_ values and metabolic acid–base conditions, FA‐based estimates showed reduced deviation from zero in both pooled and individual analyses. Similar trends were observed in the datasets of Rossing et al. (1986) and Fencl et al. (2000). Population‐based analysis by Anstey (2009), that used the Figge relationship, obtained a nonzero mean SIG of about 4 mEq/L with a rather broad SD that went into the negative region. In contrast, the much closer to zero present FA estimate of 0.58 mEq/L, coupled with similar results in the previous studies considered, favors the use of the FA relationship in all basic studies.

The SIG predictions in the study of Morgan (2009) suggest a possibly different result. Their purpose was to show that SIG estimates using the Figge formulation were stable and close to zero even with extreme PCO_2_ variation (see Table 5). Statistical analysis of their 37 measurement sets was 0.5 ± 1.5 (SD), but some of their estimates exceeded 3 mEq/L. A problem was that [Mg^2+^] was not measured so an assumed normal value was used to amend the calculations in the undiluted case and a proportionally lower value in the diluted case. The result was that models with both charge formulations were close to the theorized zero value, other than one case.

Clinical cases were included for different reasons because the patients could not be expected to have theoretical zero‐SIG values. In the cardiac‐bypass study of Morgan et al. (2016), SIG values (denoted [XA]) were approximately known at the four measurement time points (Table 6) because acetate and gluconate concentrations in the patients' blood were measured directly, reflecting their high concentrations in the bypass fluids used during the procedure. At the pre‐bypass time point (T1), the calculated [XA] value was 1.41 mEq/L, within the normal range of 0–2 mEq/L. Because Morgan et al. (2016) used the Figge model for SIG calculations, the present Figge‐model estimate would be expected to be similar. However, a major limitation of the study was that [Mg] was not measured, requiring the present calculations to assume a normal concentration of 0.8 mM (1.6 mEq/L) in the undiluted case.

As shown in Table 6, matching the present Figge‐model SIG estimate to the reported [XA] value at T1 required reducing the assumed [Mg] contribution by 0.84 mEq/L. At T2, following initiation of bypass, matching required an increase of 4.7 mEq/L, which subsequently declined to 2.6 mEq/L before bypass separation and to 1.26 mEq/L 4 h after separation. As also shown in Table 6, the required matching adjustments in [Mg] would be somewhat greater for the FA‐model estimates. These findings demonstrate the substantial effect of incomplete measurement sets on SIG estimation accuracy.

As shown in Table 7, for the first Rocktaeschel et al. study (Rocktaeschel, Morimatsu, Uchino, Goldsmith, et al., 2003), differences between their calculated SIG estimates and the presently calculated values were small. These discrepancies likely resulted from methodological differences, since they calculated SIG individually for each patient before averaging, whereas the present analysis used group mean measurement values directly in the calculations.

In the second Rocktaeschel et al. study (Rocktaeschel, Morimatsu, Uchino, & Bellomo, 2003), the authors concluded that the 1.7 mEq/L difference between groups was not statistically significant. In the present analysis, recalculated Figge‐model SIG estimates were consistently approximately 1–2 mEq/L higher in both groups, although the basis for this systematic difference remains unclear. It is possible that these small differences could affect statistical results. Interestingly, FA‐model predictions (not shown) more closely approximated the values reported by them than did the corresponding Figge‐model recalculations (Table 7). The study by Kroustalakis et al. (2025) (Table 8) found that post‐hemodialysis (HD) SIG estimates were significantly lower than those of both pre‐HD and peritoneal dialysis (PD) patients, indicating that HD effectively reduced the burden of unmeasured anions. They also concluded that the PD patients retained a substantial burden of unmeasured anions. Notably, the smaller FA‐model SIG estimates placed the post‐HD patients within the normal SIG range, whereas the Figge‐model estimates remained elevated. These findings highlight the potential value of SIG measurements in assessing acid–base status in renal failure patients and suggest that the FA formulation may provide a more clinically useful interpretation.

## 5 CONCLUSIONS

Across multiple independent normal datasets, the Fogh‐Andersen albumin charge formulation (Figge 2009) produced SIG estimates that were consistently closer to zero than those obtained using the Figge model. This improvement was observed under a range of experimental conditions, including variations in PCO_2_ and albumin concentration. The Fogh‐Andersen formulation therefore appears to provide a more appropriate basis for SIG calculations in experimental acid–base analyses. However, in clinical disease states where albumin concentrations are reduced, both formulations appear adequate for identifying clinically significant differences in SIG; but small errors in calculation could obscure these differences.

## AUTHOR CONTRIBUTIONS

**Matthew B. Wolf:** Conceptualization; data curation; formal analysis; investigation; methodology; software; supervision; validation; visualization.

## FUNDING INFORMATION

No funding information provided.

## CODE AVAILABILITY

All code is available on the Excel spreadsheets in Data S1–S6.

## ETHICS STATEMENT

No human or animals were used in this study. The only data used were from referenced publications for the purpose of constructing mathematical models of physiological systems. No AI was used in any of the scientific aspects of the study.

## Supporting information


Data S1.

Data S2.

Data S3.

Data S4.

Data S5.

Data S6.

## Data Availability

All measurement data are on Excel spreadsheets in Data S1–S6.
